# The odd one out: *Arabidopsis* reticulon 20 does not bend ER membranes but has a role in lipid regulation

**DOI:** 10.1038/s41598-018-20840-0

**Published:** 2018-02-02

**Authors:** Verena Kriechbaumer, Lilly Maneta-Peyret, Laetitia Fouillen, Stanley W. Botchway, Jessica Upson, Louise Hughes, Jake Richardson, Maike Kittelmann, Patrick Moreau, Chris Hawes

**Affiliations:** 1 Plant Cell Biology, Biological and Medical Sciences, Oxford Brookes University, Oxford, OX3 0BP United Kingdom; 2 Laboratoire Biogenèse Membranaire, UMR 5200 CNRS-Université de Bordeaux, Villenave d’Ornon, France; 3 MetaboHub-Metabolome Facility of Bordeaux, Functional Genomics Center, Bordeaux, France; 4 Central Laser Facility, Science and Technology Facilities Council (STFC) Rutherford Appleton Laboratory, Research Complex at Harwell, Didcot, OX11 0QX United Kingdom; 5 Present Address: J.U.: The Sainsbury Laboratory, Norwich, United Kingdom

## Abstract

Reticulons are integral ER membrane proteins characterised by a reticulon homology domain comprising four transmembrane domains which results in the proteins sitting in the membrane in a W-topology. Here we report on a novel subgroup of reticulons with an extended N-terminal domain and in particular on arabidopsis reticulon 20. Using high resolution confocal microscopy we show that reticulon 20 is located in a unique punctate pattern on the ER membrane. Its closest homologue reticulon 19 labels the whole ER. Other than demonstrated for the other members of the reticulon protein family RTN20 and 19 do not display ER constriction phenotypes on over expression. We show that mutants in RTN20 or RTN19, respectively, display a significant change in sterol composition in roots indicating a role in lipid regulation. A third homologue in this family -3BETAHSD/D1- is unexpectedly localised to ER exit sites resulting in an intriguing location difference for the three proteins.

## Introduction

The endoplasmic reticulum (ER) is a multifunctional organelle^1^ involved in a plethora of aspects of plant life. The polygonal network of the cortical ER consists of motile tubules that are capable of morphing into small cisternae, mainly at the three-way junctions of the ER network^2^. The plant cortical ER network has been shown to play numerous roles in protein trafficking^1,3^ and pathogen responses^4^. It is a highly dynamic organelle and previous studies have demonstrated a possible link between ER structure and function within different cell- and tissue-types^5,6^.

A variety of ER movements have been characterised, including growth and shrinkage of tubules, rearrangement of the polygonal network^2^, movement of the membrane surface^7^, and the conversion between cisternal and tubular ER^1^. These distinct movements which appear dependent on the acto/myosin system^2^ and the possibly significant link between structure and function, makes these processes important to understand.

### The reticulon protein family

Reticulons are a family of ER-localised proteins found in a wide range of eukaryotes and have been shown to localise to the ER in many species, including mammals, yeasts and plants^8,9^. Previous studies have demonstrated a role for reticulons in moving and shaping the ER into tubules^9,10^.

Plant reticulons (RTNLB-reticulon-like protein B; henceforth referred to as RTN) are considered to be essential in maintaining the tubular ER network as they contribute significantly to tubulation of the ER^10,11^. In arabidopsis, the reticulon protein family comprises 21 members^8,12^. Despite overlapping functions of the members of this protein family, variation in reticulon isoform expression does occur within different tissues. For example, AtRTN13 was found to be more abundant in seeds compared with the rest of the plant^13^, suggesting there may be cell-specific roles for RTN isoforms^9^. RTNs are integral membrane proteins characterised by a C-terminal reticulon homology domain (RHD) which has been suggested to generate and/or stabilize curvature of the membrane. This conserved domain of about 200 amino acids contains two long hydrophobic regions flanking a hydrophilic loop. The hydrophobic regions can each be further subdivided into two transmembrane domains (TMDs) resulting in a ‘W’–like topology. The N-and C-termini of the protein are facing the cytosol^13^. Reticulon proteins can dimerize or oligomerize and thereby cause localized tensions in the ER membrane inducing membrane curvature^13^. When overexpressed *in planta*, RTNs induce severe constrictions of ER tubules and are able to convert ER membrane sheets into tubules^10,11,13^. More recently AtRTN13 was shown to harbour an amphipathic helix (APH) at its cytosolic C-terminus which also appears to be involved in inducing ER membrane curvature^14^.

The membrane constriction of RTNs has also been shown to be of importance in the context of cell plate formation and primary plasmodesmata^15^. Plasmodesmata formation is dependent on tubulating the cortical ER to form the desmotubules, axial structures of approximately 15 nm diameter crossing the plasmodesmata pore and thereby connecting the ER of two cells^16,17^. Two reticulon proteins (RTN3 and RTN6) were identified in a proteomic study as plasmodesmata-localised proteins^18^ and could also be shown recently to be present in primary plasmodesmata at cytokinesis^15^. RTN3 and 6 also interact specifically with themselves and each other and a variety of plasmodesmata proteins and proteins previously described as targets of viral movement proteins^19^.

A third reticulon protein predicted to be plasmodesmata localised is RTN20 (TAIR, https://www.arabidopsis.org). RTN20 is one of 5 reticulon proteins (RTN17-21) that features an additional N-terminal domain. For RTN20 this domain is predicted to have an enzymatic function in sterol biosynthesis (AraCyc).

### Plant sterols

Plant sterols such as sitosterol, stigmasterol and campesterol influence the permeability and fluidity of membranes by lipid-lipid as well as lipid-proteins interactions within the membrane^20^. Enhanced sterol levels can often be detected in detergent-insoluble membrane rafts in the plasma membrane^21,22^ which are suggested to be important for signalling processes^23,24^ involving for example auxin transport^25^ and PAMPs (pathogen-associated molecular patterns, ref^26^.). Sterol molecules become functional after removal of the two methyl groups at the C-4 position of cycloartenol, a precursor molecule of plant sterols. The enzymatic activities required for this C-4 demethylation in plants have been characterized: this requires the activity of a sterol C-4 methyl oxidase^27,28^, a 3beta-hydroxysteroid dehydrogenase/C-4 decarboxylase (3BETAHSD/D)^29^ and an NADH-dependent 3-oxosteroid reductase^30^. One of the previously characterized 3BETAHSD/D proteins (ref^31^., 3BETAHSD/D2) is now known to be a reticulon (RTN19). By using a three-dimensional homology modeling to identify key amino acids, it has been determined that this protein is a bifunctional short-chain dehydrogenase/reductase enzyme^32,33^.

Here using high resolution confocal microscopy we show a unique punctate localisation for RTN20 on the ER membrane and the inability of RTN20 and 19 to display ER constriction phenotypes on overexpression as demonstrated by the so far published members of the family^13^. We also report that these reticulons may have a novel role in lipid regulation. Mutants in RTN20 or RTN19, respectively, display a significant reduction in sterol levels in the roots. A third homologue in this family -3BETAHSD/D1 with no transmembrane domains- is unexpectedly located to ER exit sites resulting in an intriguing location difference for the three homologues.

## Results

### Reticulon phylogeny and sub-classes

**Figure 1:**
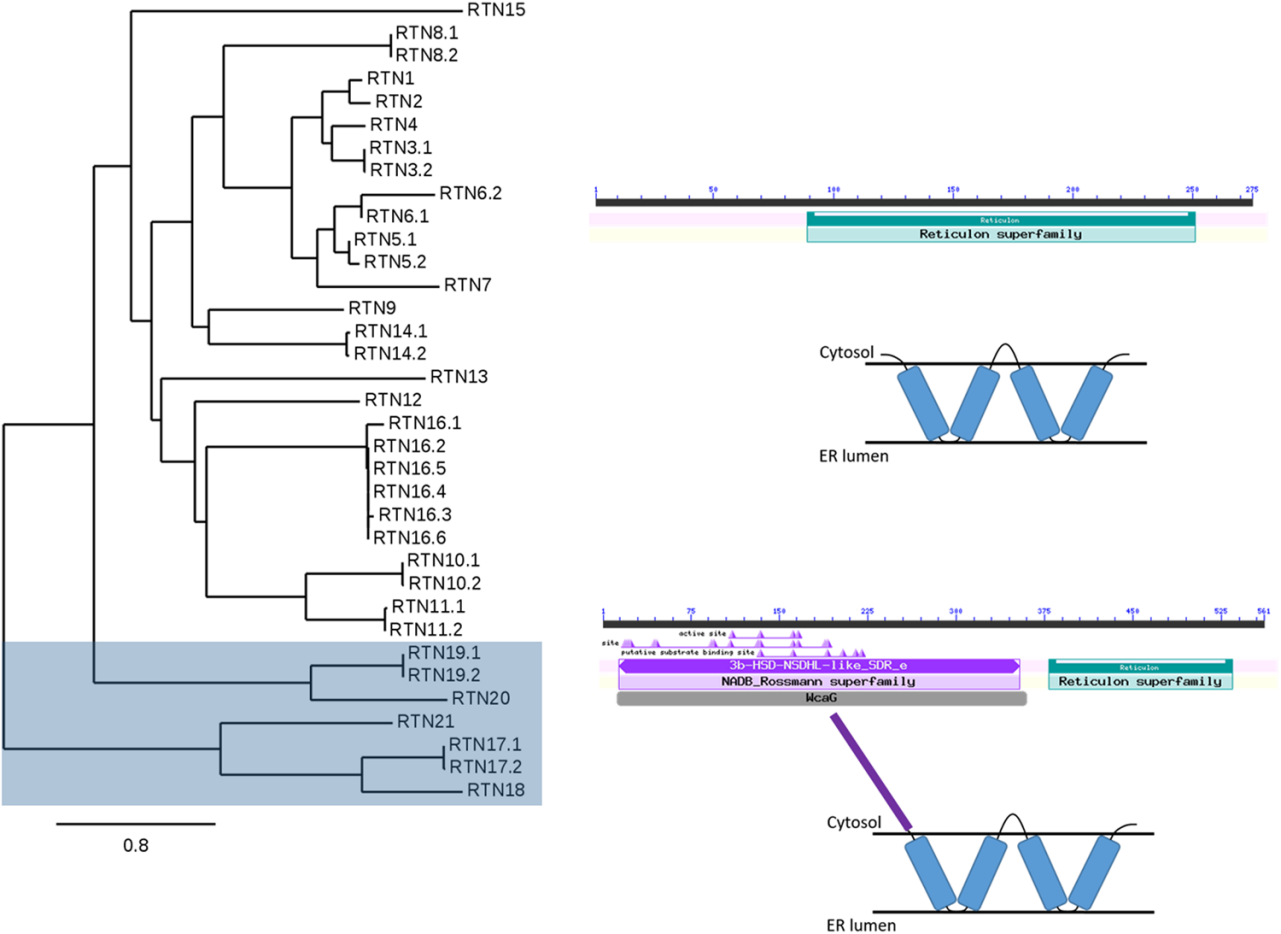
Phylogenetic analysis of reticulon proteins. Phylogenetic relationships within the arabidopsis reticulon family including alternate splice isoforms are shown. Reticulon proteins with an extended N-terminal domain (RTN17-21) are highlighted with a grey shade (left). Bar for bootstrap value is shown. BLAST domain annotations and membrane topology diagrams for this group as well as for the other reticulon family proteins (RTN1-16) are indicated (right).

The reticulon protein family in *Arabidopsis thaliana* consists of 21 members which group according to structural organisation of the functional domains, with those proteins mainly consisting of the reticulon homology domain (RTN1-16) grouping together but clearly differentiated from the reticulons with an additional N-terminal domain (RTN17-21) (Fig. 1). Within the group of reticulons containing the additional N-terminal domain RTN19 and 20 are again phylogenetically differentiated from RTN17, 18, and 21.

### RTN20 localises to punctae on the ER but lacks the reticulon constriction phenotype

**Figure 2:**
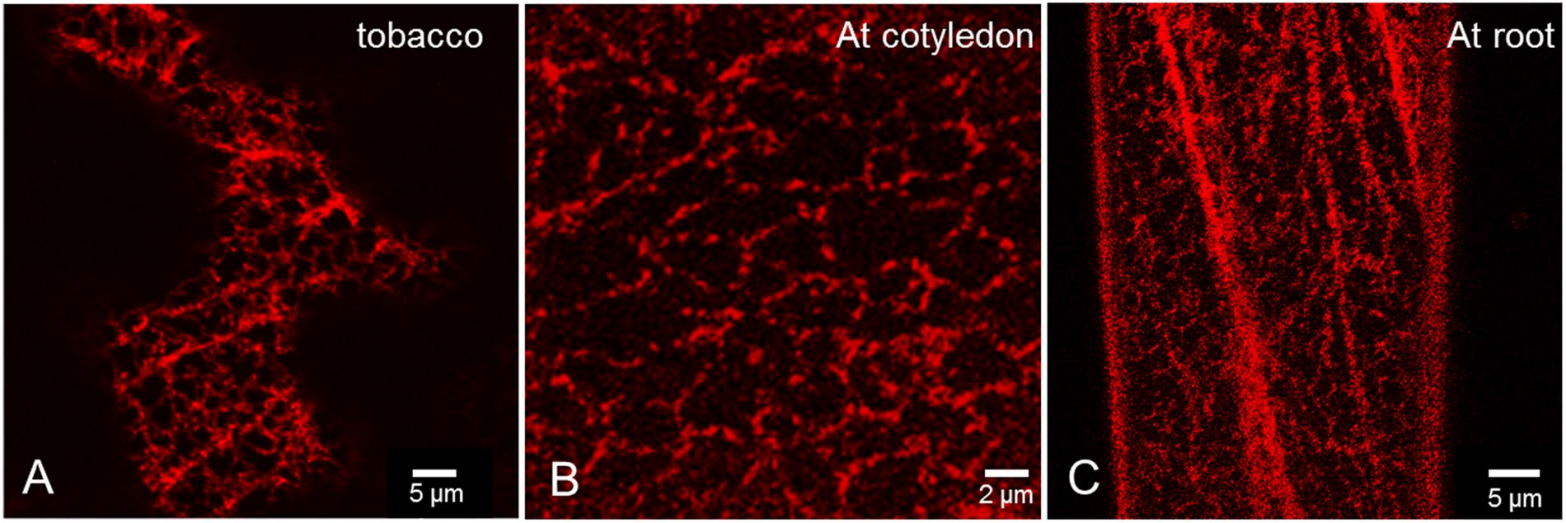
Comparison of RTN20 expression in tobacco and arabidopsis. Expression of YFP-RTN20 is shown transient in tobacco leaf epidermal cells (**A**) and in a stable manner in *Arabidopsis thaliana* cotyledon cells (**B**) and root cells (**C**).

RTN20 tagged to the yellow fluorescent protein was transiently expressed in tobacco leaf epidermal cells using *Agrobacterium*-mediated transformation (Fig. 2A). YFP-RTN20 labels the ER but rather than showing the typical ER tubule phenotype, when observed using confocal imaging, the expression pattern appears as dots along the ER (Fig. 2A). The same unusual expression pattern could be observed when RTN20 was stably transformed into *Arabidopsis thaliana* (Fig. 2B,C).

**Figure 3:**
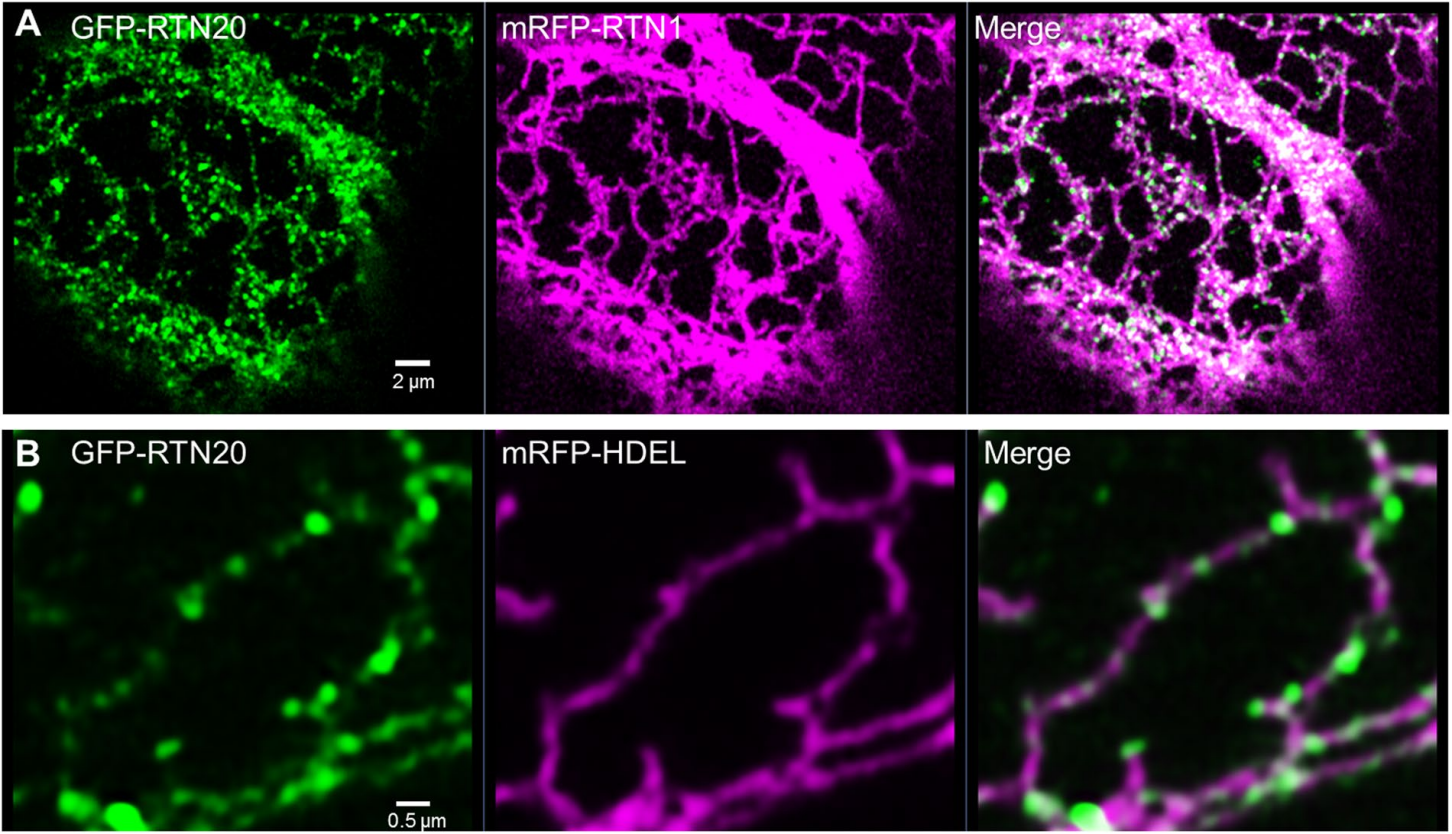
Airyscan confocal images for RTN20 subcellular localisation. GFP-RTN20 is expressed in tobacco leaf epidermal cells by *Agrobacterium*-mediated transient expression together with mRFP-RTN1 (**A**) or mRFP-HDEL (**B**), respectively. Size bars are given.

High resolution Airyscan imaging of GFP-RTN20 co-expressed with mRFP-RTN1 clearly shows the punctate structure of RTN20 on the ER membrane (Fig. 3A). Interestingly RTN20 does not exhibit the typical reticulon constriction phenotype reported for all previously described reticulons^13,14^ when coexpressed with GFP-HDEL (Fig. 3B). Hence RTN20 is assumed to have a different function other than tubulating the ER.

**Figure 4:**
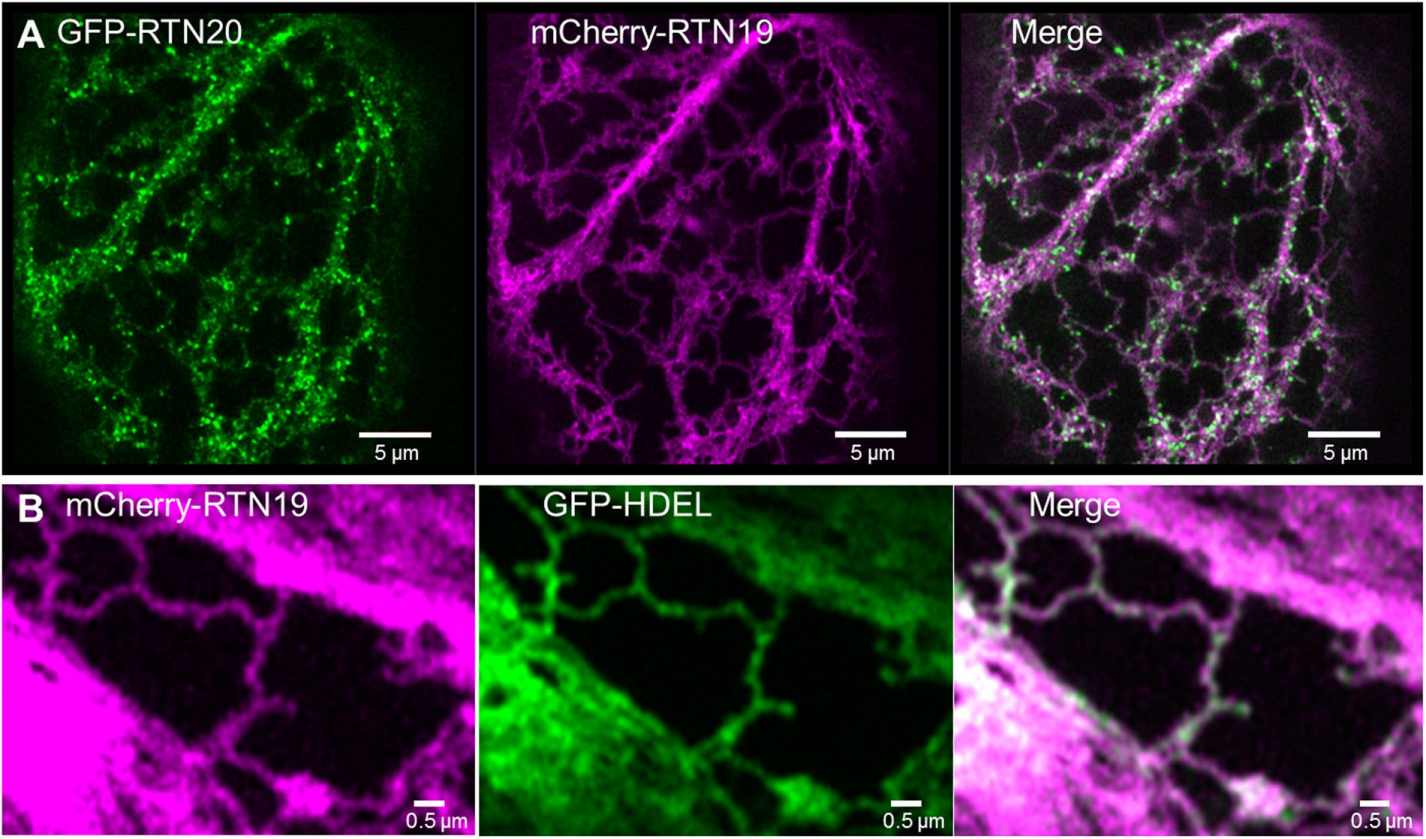
Confocal images for RTN19 localisation. RTN19 fused to mCherry is coexpressed in tobacco leaf epidermal cells with RTN20 (top row) as well as the ER-marker GFP-HDEL (bottom row). RTN19 shows co-localisation with HDEL but not the dotted pattern of RTN20. Size bars are given.

To determine if the localisation of RTN20 is a feature for the group of reticulons with extended N-terminal domain, subcellular localisation of RTN19 was also tested as this is the most closely related reticulon to RTN20. RTN20 and RTN19 share 42% amino acid identity. In confocal imaging RTN19 clearly labels the ER when co-expressed with either RTN20 or GFP-HDEL, respectively. It shows classic ER membrane labelling as other reticulons^2^ rather than the punctate expression pattern along the ER of RTN20 (Fig. 4A), but again does not produce the constriction phenotype described for other reticulons (Fig. 4B).

To test the cause of this difference in localisation between RTN20 and RTN19, fusions between the N-terminus of RTN19 and the C-terminus of RTN20 were created (Supplementary Fig. S1). Fusing the RTN20 C-terminus including the transmembrane domain area as well as the C-terminal tail to the N-terminus of RTN19 (lacking its TMDs) resulted in RTN19 localising to punctate structures similar to RTN20 localisation (Supplementary Fig. S1A). The same change in localisation happened when only the C-terminal tail without the TMDs from RTN20 was fused to the N-terminus of RTN19 with its TMD (Supplementary Fig. S1B). It is therefore very likely that the punctate distribution of RTN20 is due to sequence differences at the C-terminus in the region of the APH which can also be linked to the lack of a constriction phenotype with RTN20.

In order to assess whether the punctate distribution of YFP-RTN20 had any effect on overall ER morphology, we carried out an electron microscope analysis of ER structure in YFP-RTN20 expressing arabidopsis plants and in wild type plants. As leaf cells are very large and therefore difficult to use for EM analysis of cortical ER, and the role of the protein in sterol regualtion may be restricted to roots (see below), we used root tip cells as our experimental material. To visualise the ER network, the ER was selectively stained using the zinc iodide osmium tetroxide impregnation technique and reconstructed in 3-D by serial block-face scanning electron microscopy^34^. No major differences were observed in ER structure between wild type and YFP-RTN20 expressing plants (Supplementary Fig. S2) suggesting that the punctate distribution of fluorescence most likely reflects clustering of the protein in patches on the ER membrane. Also despite the root lipid phenotype in the *rtn20* mutant plants no significant differences could be observed in mutant roots at the EM level (Supplementary Fig. S2).

### RTN 20 interacts with other reticulon proteins

It has been shown that reticulon proteins are capable of forming homomers as well as dimers with other reticulon proteins^13^. Due to the difference in the N-terminal domain structure, it was of interest to test if RTN20 is still capable of such interactions. Förster resonance energy transfer by fluorescence lifetime imaging microscopy (FRET-FLIM) analysis to confirm prey-bait interactions *in vivo* was applied^19^. This technique enables the measurement of the space map of picosecond fluorescence decay at each pixel of the image through confocal single and multiphoton excitation. Förster resonance energy transfer (FRET) was used to define physical interactions of protein pairs tagged with appropriate GFP fluorophores and monomeric red fluorescent protein (mRFP).

In case of protein-protein interactions FRET-FLIM shows reductions in the excited-state lifetime of GFP (donor) fluorescence in the presence of the acceptor fluorophore (mRFP). A reduction in fluorescence lifetime of the donor indicates that the two tested proteins are within a distance of 10 nm or less and thereby indicating a physical interaction between the two proteins^13^.

Due to limitations in the speed of photon counting of the FLIM set-up, measurements were taken from high-expressing regions of ER showing relatively low mobility, such as the ER associated with the nuclear envelope. This allows more reliable measurements than the fast-moving cortical ER^13^ and the use of actin depolymerising agents such as Latrunculin B which will perturb ER structure can be avoided.

**Figure 5:**
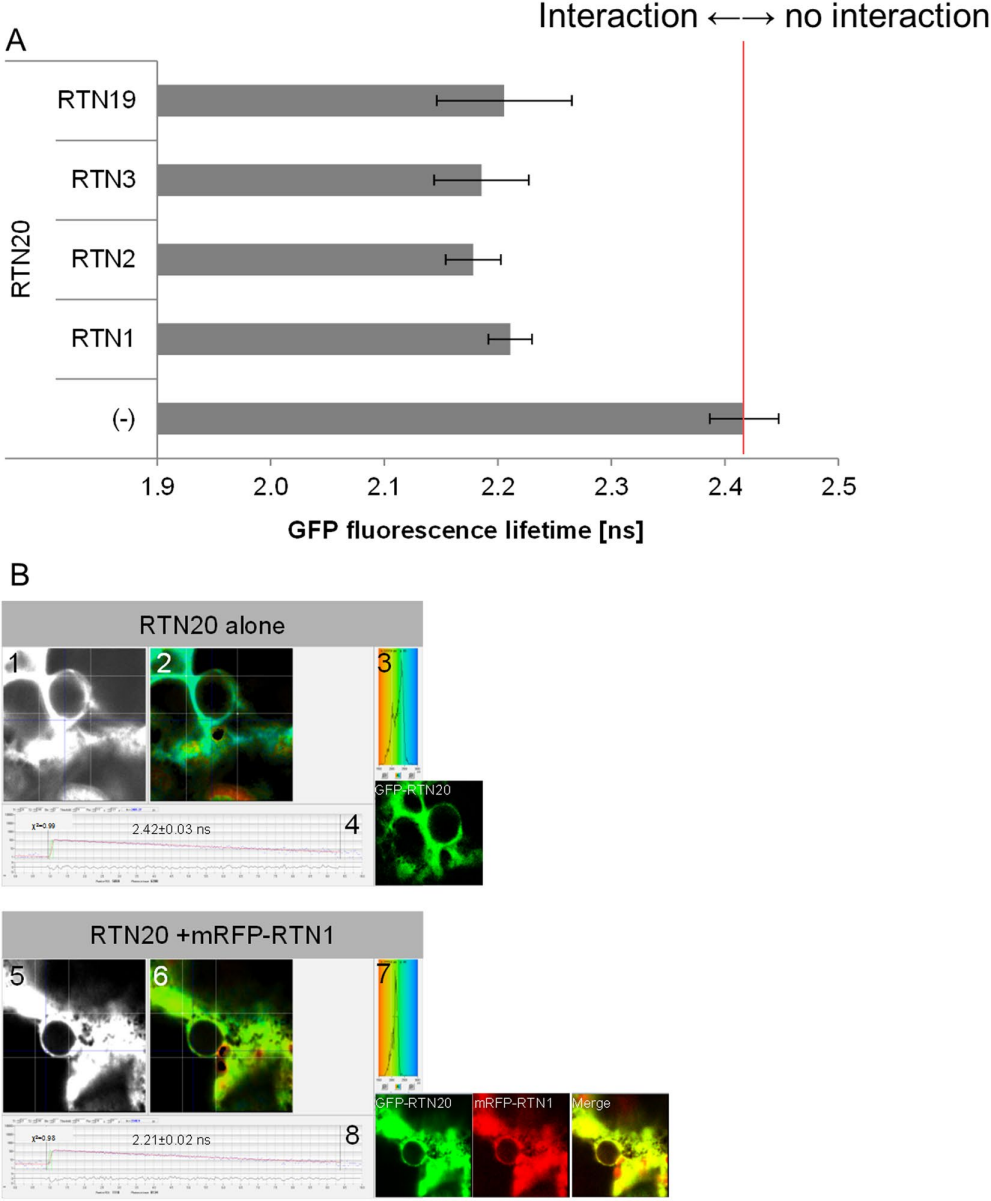
RTN20 protein interactions measured by FRET-FLIM. (**A**) Bar chart of fluorescent lifetimes for the donor GFP-RTN20 alone as a negative control and in heteromerisation with RTN1, 2, 3, 19 fused to mRFP as acceptor. (**B**) FRET-FLIM analysis of RTN20. FRET-FLIM analysis of RTN20 without an interaction partner^1–4^ or RTN20-RTN1 dimerization^5–8^ is shown. 1 and 5 show the raw FRET-FLIM data. In 2 and 6, pseudo-coloured lifetime maps display the lifetime values for each point within the region of interest. The distribution of lifetimes across the entire image is shown in 3 and 7 with blue shades representing longer GFP fluorescence lifetimes than green ones. 4 and 8 show representative decay curves of a single point with an optimal single exponential fit, where χ^2^ values from 0.9 to 1.2 are considered an excellent fit to the data points. Respective confocal images for the analysis are given with the GFP construct in green and the mRFP construct in red. This example of FRET-FLIM analysis shows that RTN20interacts with RTN1 because the lifetime values for the GFP/mRFP fusion pair (8; 2.21 ± 0.02 ns) are lower than those for the GFP fusion alone (4; 2.42 ± 0.03 ns).

Here, GFP-RTN20 was used as a donor and the reticulon proteins RTN1, 2, 3, and 19 were fused to mRFP as acceptor proteins. These reticulons were chosen to represent the various sub-groups of reticulons known to date: RTN1, 2, and 3 feature the typical reticulon domain topology whereas RTN19 features an additional N-terminal enzymatic domain similar to RTN20. Furthermore RTN3, together with RTN6, has been shown to be localised to plasmodesmata^15^. For each combination at least two biological samples with a minimum of three technical replicates each were used for the statistical analysis. FRET-FLIM interaction results are shown in Fig. 5 and Supplementary Figure S3. Live cells expressing RTN20 alone without an acceptor present was used as a control and resulted in a baseline fluorescence life time of 2.4 ± 0.03 ns. Excited-state lifetimes determined for all RTN-RTN heteromeric interactions tested showed an average fluorescence lifetime of 2.2 ns (Fig. 5A, Supplementary Fig. S3) which is 0.2 ns lower than the donor alone and statistically significantly different from that of the GFP alone.

Figure 5B shows the FRET-FLIM analysis steps for GFP-RTN20 alone (Fig. 5B1–4) as a control and for GFP-RTN20 interacting with mRFP-RTN1 (Fig. 5B5–8) as an example for interaction. Raw FRET-FLIM images are shown in Fig. 5B1 and 5. This analysis takes into account the lifetime values of each pixel within the image visualized by a pseudocoloured lifetime map (Fig. 5B2, 6). The graph shows the distribution of lifetimes within the image (Fig. 5B3, 7), with blue shades representing longer GFP fluorescence lifetimes than green/yellow ones. Decay curves (Fig. 5B4, 8) of a representative single pixel highlight an optimal single exponential fit, where χ^2^ values in the range of 0.9 to 1.2 were considered an excellent fit to the data. Confocal images for the region of interest showing the GFP construct in green and the mRFP construct in red are shown.

This specific example (Fig. 5B) shows that RTN20 heterodimerizes with RTN1 as the lifetime values for the GFP/mRFP fusion pair (2.21 ± 0.02 ns; Supplementary Fig. S3B) are significantly lower than those for the GFP fusion alone (2.42 ± 0.03 ns).

### Lipid analysis: *rtn20* mutants show decreased sterol levels

As the N-terminus of RTN20 is predicted to be involved in sterol biosynthesis, lipid composition in the *rtn20* mutant was tested. The closest homologue to RTN20 -RTN19- has previously been shown to possess 3beta-hydroxysteroid dehydrogenase/C-4 decarboxylase (3BETAHSD/D) activity^31^ and the *rtn19* mutant was therefore included in the lipid analysis. Furthermore, a mutant rescue line was created by overexpressing RTN20 under a 35 S promoter in the *rtn20* mutant background.

**Figure 6:**
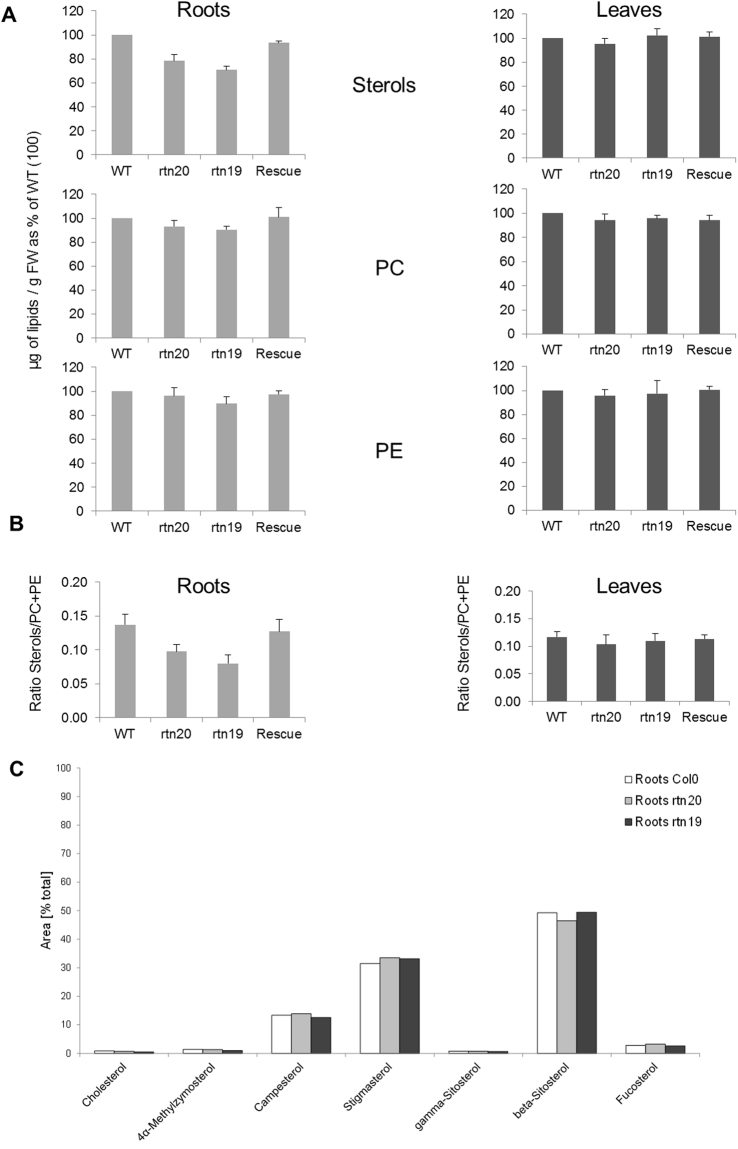
Lipid composition of the reticulon mutants *rtn19* and *rtn20* and rescue lines. (**A**) Sterols and the major phospholipids phosphatidylcholine (PC) and phosphatidylethanolamine (PE) from roots and leaves of two week-old seedlings (wild type, mutant lines *rtn20* and *rtn19*, and rescue line) were quantified. Data was obtained from 3 independent experiments by HPTLC coupled to densitometry. Sterol, PC and PE amounts were determined as µg/g FW, the values for the control roots and leaves were normalized to 100 and the corresponding values for the mutant and rescue lines were calculated accordingly. (**B**) The ratios between sterols to PC + PE content are indicated for the different lines.

Sterols and the major phospholipids phosphatidylcholine (PC) and phosphatidylethanolamine (PE) from roots and leaves of two-week-old seedlings (wild type, mutant lines *rtn20* and *rtn19*, and rescue line) were determined from 3 independent experiments by HPTLC coupled to densitometry. The level of sterols, PC and PE in the roots and leaves of control, mutant and rescue lines were determined as µg/g FW, and the values for the control roots and leaves were taken as equal to 100 and the corresponding values for the mutant and rescue lines were calculated accordingly (Fig. 6A). The ratios sterols to PC + PE are also indicated for the different lines (Fig. 6B).

We determined the following amounts of sterols, PC and PE in the control roots (243 ± 28 µg/g FW of sterols; 886 ± 74 µg/g 74FW of PC and 762 ± 81 µg/g FW of PE) and control leaves (188 ± 23 µg/g FW of sterols; 991 ± 86 µg/g FW of PC and 547 ± 48 µg/g FW of PE). A significant decrease of the level of sterols was observed in the roots of the *rtn20* and *rtn19* mutants (p < 0.01) but no variation of sterols was observed in the leaves of the *rtn20* and *rtn19* mutants, and no significant variation of the phospholipids PC and PE was observed both in the roots and the leaves of the *rtn19* and *rtn20* mutants (Fig. 6A). This also led to a decrease of the sterols to phospholipid (PC + PE) ratio in the roots of the *rtn20* and *rtn19* mutants (Fig. 6B; p < 0.01). Expressing the RTN20 protein in the *rtn20* mutant background restored the level of sterols (Fig. 6A; p > 0.2) and the sterols to phospholipids (PC + PE) ratio in the roots of the rescue lines (Fig. 6B; p > 0.5). This reduction could indicate a direct role for RTN20 in lipid synthesis most likely via a 3beta-hydroxysteroid dehydrogenase/C-4 decarboxylase (3BETAHSD/D) activity as shown previously for RTN19^31^ but also a regulatory role. To further investigate if this reduction in sterol content is due to a reduction in specific sterol species and to further elucidate the function of RTN20 and RTN19, we carried out GC-MS analysis to quantify sterol composition in roots of Col0, *rtn20* and *rtn19* in more detail. GC-MS analysis for wild type and mutant plants revealed no significant differences for any of the analyzed sterol species or overall sterol composition. These results suggest that RTN20 is probably not directly involved in lipid biosynthesis in arabidopsis roots but more likely has a role in regulating the *de novo* bulk synthesis in the ER.

**Figure 7:**
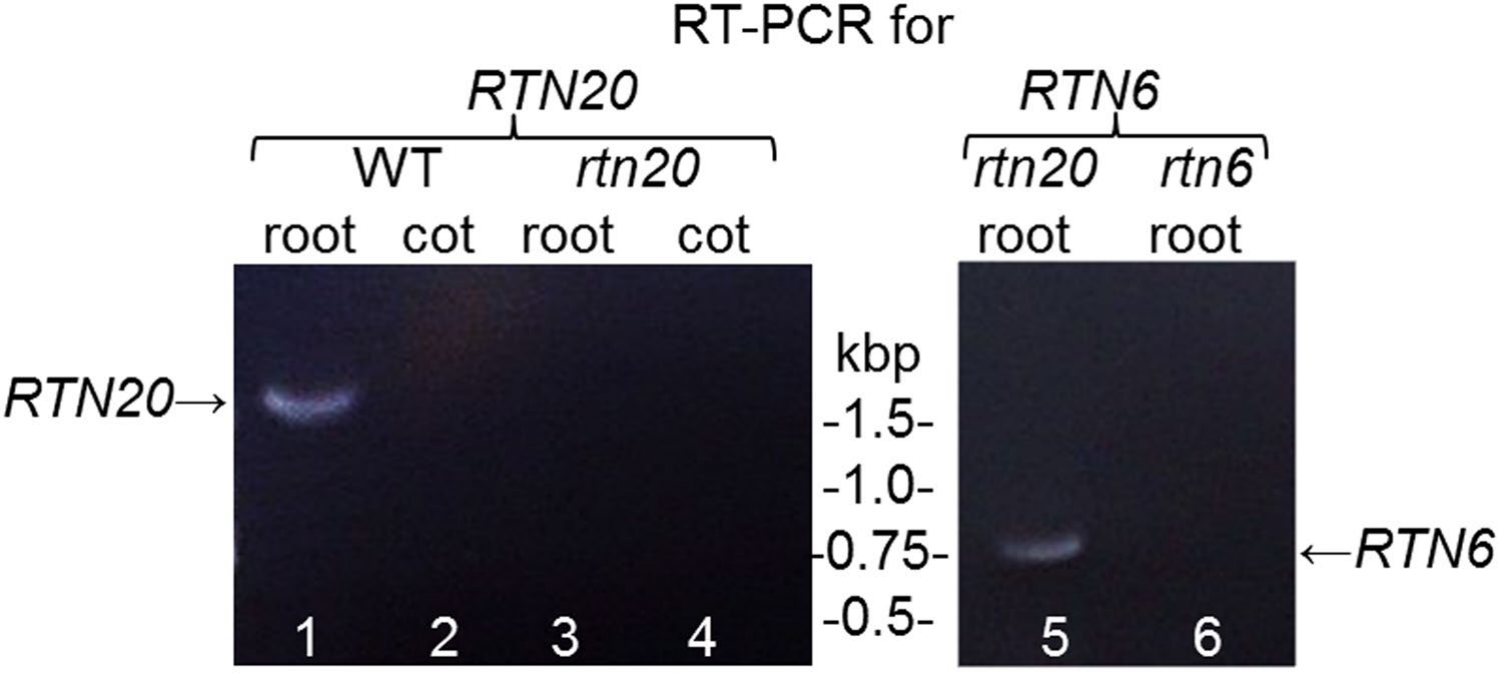
Transcription of RTN20. Reverse transcriptase PCR was performed in wild type (WT) Col0 plants as well as *rtn20* and *rtn6* mutant plants. It was also distinguished between root and cotyledon (cot) tissue. *RTN20*-mRNA was detectable in WT roots but not in cotyledon tissue or in the *rtn20* mutant. *RTN6* could be detected in the *rtn20* mutant but not in the *rtn6* mutant.

As the mutant lipid testing showed different effects between roots and leaves *RTN20* transcript levels were examined. To test for the presence of *RTN20* mRNA reverse transcriptase PCR was performed (Fig. 7, Supplementary Figure S4) using root and cotyledonary tissue from both wild type (WT) Col0 plants and *rtn20* mutant seedlings. *RTN20* transcript could be detected in wild type root tissue but not in cotyledonary tissue (Fig. 7, lane 1 and 2) which could account for the change in lipid composition in *rtn20* roots but not in cotyledonary tissue at this developmental stage. *rtn20* mutant plants showed no detectable *RTN20* transcript (Fig. 7, lane 3 and 4). To test for the quality of the cDNA *rtn20* mutants were also probed with primers for *RTN6* resulting in a *RTN6* band (Fig. 7, lane 5) that was not detectable in the *rtn6* mutant background (Fig. 7, lane 6).

### “The third man”: 3BETAHSD/D1, a RTN20 homologue, localises to ER exit sites

Phylogenetic analysis revealed that arabidopsis RTN20 is highly homologous to the yeast protein Erg26p and the mammalian protein sterol-4-α carboxylate 3-dehydrogenase (Supplementary Fig. S5); both of these analogous proteins have a role in sterol biosynthesis^35^. Additionally, these proteins were found to be homologous to the arabidopsis 4-α carboxysterol-C3-dehydrogenase (3beta-hydroxysteroid-dehydrogenase/ decarboxylase isoform 1, 3BETAHSD/D1; 31), also involved in sterol biosynthesis. Phylogenetic analysis demonstrated that the newly identified proteins all grouped with RTN19 and RTN20 rather than with the other reticulon proteins with an additional N-terminal domain (RTN17, 18, 21) (Supplementary Fig. S5), indicating these reticulons may have similar functions. 3BETAHSD/D1 shows 46% amino acid identity with RTN20 and 82% identity with RTN19 (BLASTP).

3BETAHSD/D1 is not predicted to have any hydrophobic transmembrane domains (Supplementary Fig. S6) or a signal peptide, but features a potential ER retrieval signal at the C-terminus (KKID, 31). Transformation of the yeast ergosterol *erg26* mutant, which lacks 3BETAHSD/D activity, with the arabidopsis 3BETAHSD/D1 gene can complement the mutation and interestingly the activity can be found in microsomal extracts; cytosolic fractions failed to show activity, leading to the speculation that the enzyme is membrane-bound^31^. Disruption of ERG26 is lethal, and the *erg26* strain requires ergosterol or cholesterol supplementation for viability, pointing to a role in sterol biosynthesis for 3BETAHSD/D1^35^. Neither single nor double knockout plants of RTN19 and/or 3BETAHSD/D1 display a visible phenotype^36^.

**Figure 8:**
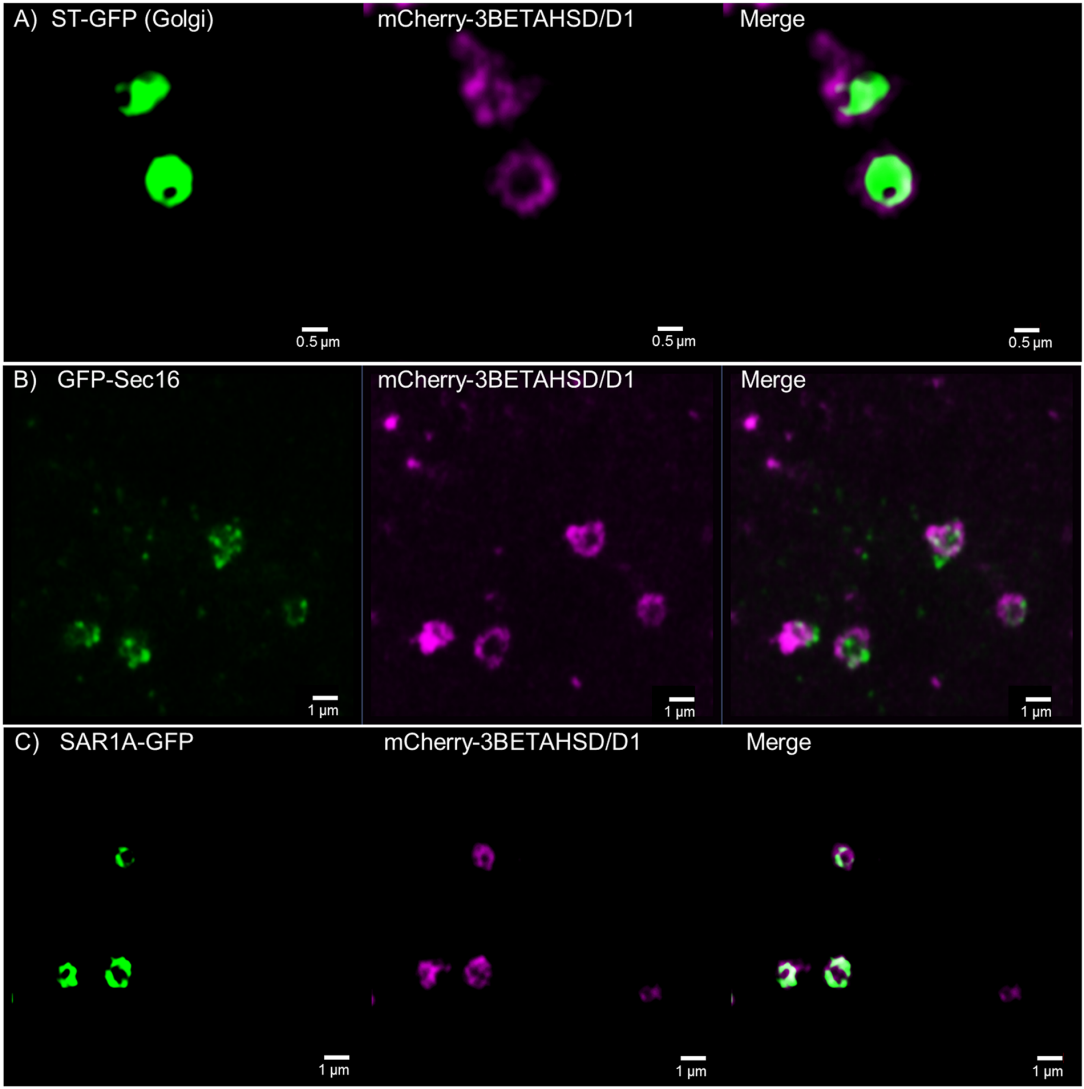
Confocal images for 3BETAHSD/D1 subcellular localisation. 3BETAHSD/D1 fused to the mCherry-fluorescent protein is coexpressed in tobacco leaf epidermal cells with the Golgi marker ST-GFP (**A**) as well as the ER exit site markers GFP-Sec16 (**B**) and SAR1A-GFP (**C**). 3BETAHSD/D1 shows co-localisation with both ER exit site markers but resembles with the ring-like structure more the SAR1A pattern than the dottier Sec16. 3BETAHSD/D1 also partially colocalises with the Golgi marker but circles the marker. Size bars are given.

When transiently expressing 3BETAHSD/D1 fused to the red fluorescent protein mCherry in tobacco epidermal leaf cells, the fusion protein showed neither cytosolic or ER localisation but distinct doughnut shaped structures around 1 micron in diameter (Fig. 8). To determine the subcellular location of these structures, mCherry-3BETAHSD/D1 was co-expressed with markers labelling Golgi bodies or ER exit sites, respectively (Fig. 8). Imaging with a high resolution Airyscan detector shows that 3BETAHSD/D1 only partially colocalises with the *trans-*Golgi marker ST-GFP and displays more of a doughnut pattern with the Golgi construct in the middle (Fig. 8A). However, 3BETAHSD/D1 colocalises with ER exit site markers AtSec16 (Fig. 8B) and AtSAR1A (Fig. 8C) all constructs labelling punctate ring-like structures. 3BETAHSD/D1 does not show any co-localisation with the RTN20 punctate structures or with the ER (Supplementary Fig. S7). We therefore conclude that 3BETAHSD/D1 resides on ER exit sites, although the exact location of such structures remains unclear.

### *3betahsd/d1* mutants show increased sterol levels

**Figure 9:**
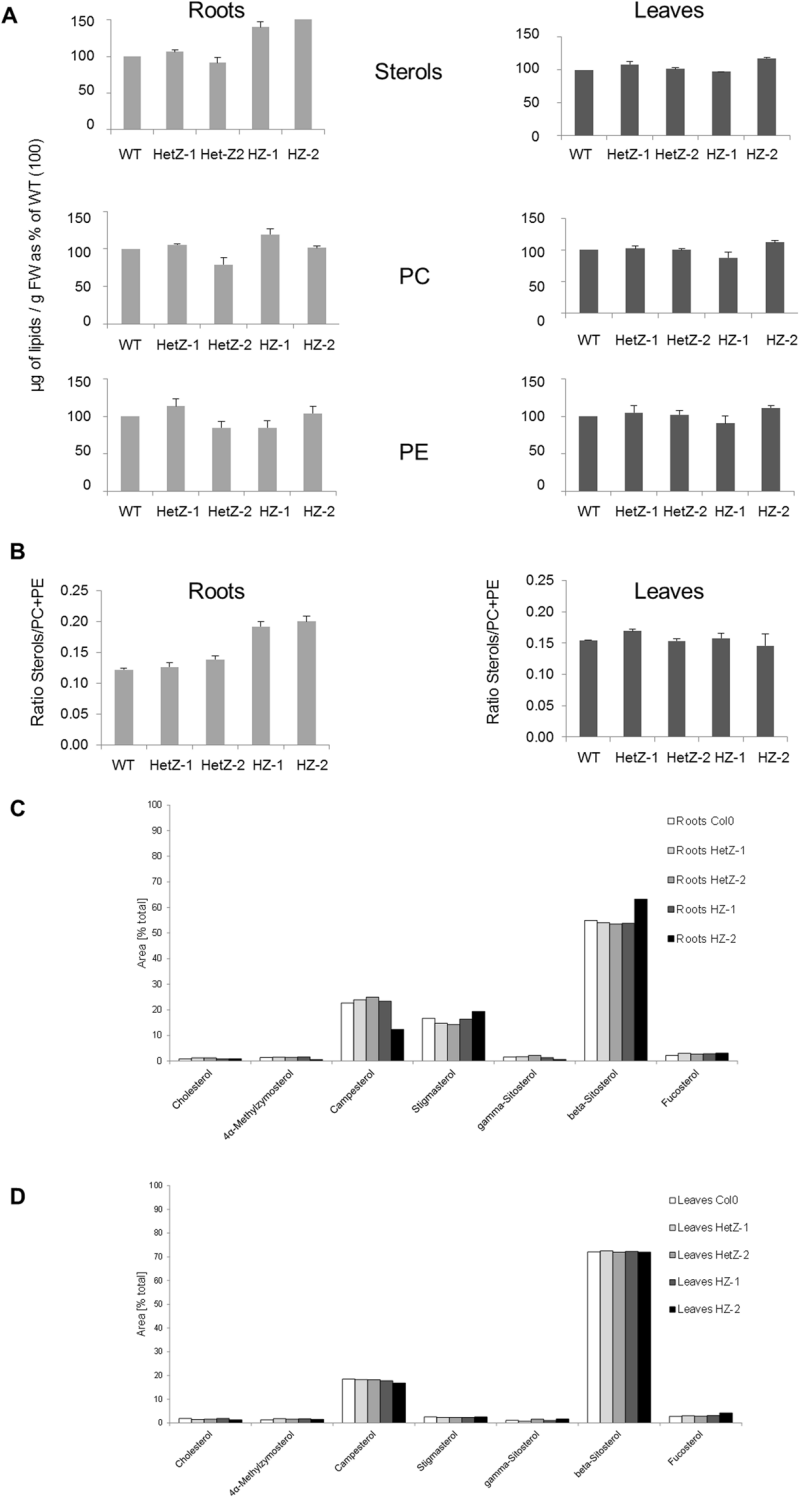
Lipid composition of *3betahsd/d1* mutant lines. (**A**) Sterols and the major phospholipids phosphatidylcholine (PC) and phosphatidylethanolamine (PE) from roots and leaves of two week-old seedlings were quantified. The lines used are wild type Col0, two lines heterozygous for *3betahsd/d1* (HetZ-1 and HetZ-2) and two lines homozygous for *3betahsd/d1* (HZ-1 and HZ-2). Data were obtained from 3 independent experiments by HPTLC coupled to densitometry. Sterol, PC and PE amounts were determined as µg/g FW, the values for the control roots and leaves were normalized to 100 and the corresponding values for the mutant and rescue lines were calculated accordingly. (**B**) The ratios between sterols to PC + PE content are indicated for the different lines. Using GC-MS different sterol species (cholesterol, 4α-methylzymosterol, campesterol, sigmasterol, gamma-sitosterol, beta-sitosterol, fucosterol) were further quantified in roots (**C**) and leaves (**D**) of two week-old seedlings of the wild type control Col0 and heterozygous (HetZ-1 and HetZ-2) and homozygous (HZ-1 and HZ-2) lines for *3betahsd/d1*. Data are presented as percentage of the overall composition.

To compare potential functions of 3BETAHSD/D1 with RTN20 sterols and the main phospholipids PC and PE were analysed in root and leaf tissue of two-week-old seedlings comparing the wild type Col0 with two lines heterozygous and homozygous, respectively, for 3BETAHSD/D1. Data were obtained from 3 independent experiments by HPTLC coupled to densitometry and expressed in µg/g FW; the values for Col0 in roots and leaves were taken as equal to 100 and the corresponding values for the mutant lines were calculated accordingly (Fig. 9A). The ratios sterols to PC + PE are also indicated for the different lines (Fig. 9B).

The following quantities of sterols, PC and PE were determined in control roots (223 ± 27 µg/g FW of sterols; 949 ± 36 µg/g 74FW of PC and 894 ± 87 µg/g FW of PE) and control leaves (220 ± 21 µg/g FW of sterols; 912 ± 16 µg/g FW of PC and 606 ± 16 µg/g FW of PE). In contrast to *rtn20* and *rtn19*, a significant increase of the level of sterols in the roots of the *3betahsd/d1* homozygous mutants is observed (p < 0.01). As for the reticulon mutants the sterol content does not change in the leaves of *3betahsd/d1* mutants, and no significant variation of the phospholipids PC and PE was observed in roots or leaves of *3betahsd/d1* mutants (Fig. 9A). Due to this we also observed a significant increase of the sterols to phospholipid (PC + PE) ratio in the roots of the *3betahsd/d1* homozygous mutants (Fig. 9B; p < 0.01). The two lines heterozygous for *3betahsd/d1* did not show any significant differences compared to the wild type Col0 (Fig. 9A,B).

GC-MS analysis of the sterol composition in roots (Fig. 9C) and leaves (Fig. 9D) was applied to this set of wild type and mutant plants. Also, similar to the *rtn20* and *rtn19* mutants, no significant changes in the sterol composition or levels of different sterol species could be detected (Fig. 9C,D). Given the difference in mutant phenotypes between the *rtn20* and *rtn19* mutants and the homozygous *3betahsd/d1* mutants we could hypothesize that a regulatory function for these proteins is specific to their localisation. Potentially 3BETAHSD/D1 could regulate sterols in the ERES in relation to membrane transport forward to the Golgi.

## Discussion

### Phylogeny and conserved domains in the reticulon protein family

Within the 21 reticulon family members in arabidopsis, five proteins cluster together due to an additional N-terminal domain from the reticulon homology domain that all reticulon proteins feature (Fig. 1). The significance of the reticulon homology domain and the C-terminal domain for the ability of reticulon proteins to induce membrane curvature has previously been demonstrated^13,14^. The reticulon homology domain has been shown to be required for reticulon residence in high-curvature ER membranes and ER tubule constriction, yet it is not necessary for reticulon oligomerisation^13^. A predicted amphipathic helix (APH) at the C-terminus of RTN13 has also been shown to be responsible for tubule constriction^14^. Upon deletion or disruption of the hydrophobic face of this APH region RTN13 loses the ability to induce constrictions of ER tubules *in vivo* but is still capable of interaction and to form low-mobility oligomers in the ER membrane^14^. However, many interactions of the cytosolic N-terminal domains are still unknown, and it is possible they confer additional functions to the proteins^10^. Within the reticulons with an extended N-terminal domain (RTN17-21) only RTN19 and 20 have domains that are predicted to be involved in sterol biosynthesis. RTN20 and RTN19 feature a predicted 3BETAHSD/D domain with a putative decarboxylating sterol-4-alpha-carboxylate 3-dehydrogenase activity (BLASTP, AraCyc) indicating a role in sterol regulation. For RTN17, 18, and 21 no conserved domain is predicted at the N-terminus (BLASTP). Reticulons in yeast have been shown to interact with other proteins, for example dynamin-related GTPases^37^, and the ability of the RTN N-terminal functional domain to facilitate protein-protein interactions may allow the reticulons to tether such molecules to the ER membrane^8^. This could be a potential role for the extended N-terminus of reticulons RTN17, 18 and 21 for which no functions have yet been predicted or identified.

### Subcellular location and protein expression pattern

All plant reticulons described so far are preferentially associated with ER tubules and the curved edges of cisternae. Overexpression of a number of reticulons results in severe constrictions of ER tubules and reticulons are able to convert ER membrane sheets into tubules^10,11,13^.

RTN20 displays a rather uncharacteristic expression pattern by labelling what appear to be ER-membrane subdomains, which can be clearly visualised *in vivo* with high resolution confocal detectors (Fig. 3). In contrast to many other reticulons including its closest homologue RTN19, RTN20 does not feature a dilysine (KKXX) ER retrieval motif at the very C-terminus. This motif promotes the retrieval of type I membrane proteins from the Golgi apparatus back to the ER^38,39^. It was however previously reported that ER retrieval motifs are non-functional when introduced near an APH region in the cytoplasmic tail^38^. For RTN20 the C-terminal region after the TMDs consists of a significantly higher ratio of hydrophobic amino acids (49%) than for RTN19 which only has 32% of hydrophobic amino acids. This results in a strong hydrophobic face for RTN20 rather than an APH present in other reticulons e.g. RTN13^14^ with a hydrophobic face opposite a hydrophilic face. As chimeric proteins with a RTN19 N-terminus and a RTN20-C-terminus display a punctate expression pattern similar to RTN20 (Supplementary Fig. S1), this RTN20 expression pattern is most likely due to elements in the C-terminal domain including the lack of an APH and ER retrieval motifs. Additionally, the lack of an APH could also account for the lack of tubule constriction ability with RTN20 indicating a different role for this reticulon rather than shaping of the ER in the way other reticulons do.

An unexpected outcome from this work was that 3BETAHSD/D1 colocalises with the ER exit site markers Sec16 and SAR1A, forms a highly structured ring of protein which locates with the classic ST-GFP Golgi marker and does not label the ER (Fig. 8). 3BETAHSD/D1 does not have any predicted ER signal sequences or TMDs but, similar to some of the reticulon proteins, features a putative di-lysine ER retrieval motif, which due to the protein location at the ERES we assume to be non-functional. ER exit sites (ERES) are specialized regions of the ER where secretory proteins are concentrated and leave the ER for export to the Golgi bodies^40,41^. They are characterized by local accumulations of COPII proteins such as Sec16, together with the dimers Sec23/24 and Sec31/13^42–44^. The localisation of 3BETAHSD/D1 on ERES is consistent with the finding of enzymatic activity for this enzyme in yeast microsomal fractions but not cytosolic fractions^31^, although the targeting to the ERES is unclear as 3BETAHSD/D1 lacks any TMDs. This could indicate that 3BETAHSD/D1 is only peripherally associated with the ERES and instead tethered via interactions with other ER-associated proteins^35^.

### A function for RTN20 in sterol regulation

Phytosterols play major roles in plants not only as structural membrane molecules^20^ but also in plant growth and development^45^ and hormone signalling^23,24,46,47^. For the biological function of these sterols the removal of the two C-4 methyl groups is crucial. It has been hypothesised that ER tubules are sites for lipid production^48^, and our mutant data strongly indicates an involvement of RTN20 in lipid regulation (Fig. 6). As the decrease in the overall sterol content in the *rtn20* and *rtn19* mutants (Fig. 6A) could not be explained by reduction in a specific sterol species (Fig. 6C) this data points more to a role in sterol regulation rather than synthesis.

### The 3BETAHSD/D domain

RTN20 features a predicted 3BETAHSD/D domain with a predicted decarboxy lating sterol-4-alpha-carboxylate 3-dehydrogenase activity (BLASTP, AraCyc). Previously, *in vitro* assays showed high 3BETAHSD/D2 activity for RTN19 (not then identified as a reticulon) and 3BETAHSD/D1 with a wide range of steroid substrates and VIGS-mediated gene silencing of RTN19 and 3BETAHSD/D1 in tobacco resulted in a significant accumulation of 3-hydroxy-4,14-dimethyl-5-ergosta-9,19-cyclo-24(241)-en-4-carboxylic acid also consistent with a decrease in 3BETAHSD/D activity^31^. Additionally, overexpression of RTN19 or 3BETAHSD/D1, respectively, in a yeast *erg26* mutant background complemented the mutant^31^, all pointing towards a role in sterol biosynthesis and/or regulation.

*3betahsd/d1* and *rtn19* single as well as double mutants display no visible phenotype during the plant life cycle^36^ indicating that other proteins such as RTN20 may be capable of carrying out the same function in sterol biosynthesis. On the other hand, overexpression of 3BETAHSD/D1 and RTN19 appears to affect auxin transporter activity and shows reduced responsiveness to the auxin efflux inhibitor NPA^36^; it is discussed that this again could be due to alterations in the sterol composition in the membranes in these overexpression lines^36^. Overall our data shows that *rtn20* and *rtn19* mutants display significant reduction in the sterol content of arabidopsis roots (Fig. 6A,B), whereas *3betahsd/d1* mutants show increased sterol levels (Fig. 9A,B). This could either indicate a localisation-specific enzymatic function of the predicted N-terminal enzymatic domain in sterol biosynthesis or a more general regulatory function. Detailed analysis of the sterol composition in these mutants did not reveal any significant up or downregulation of specific sterol species (Figs 6C and 9C) which would rather indicate a role in regulation of *de novo* bulk synthesis of sterols.

Furthermore, *rtn20* and *rtn19* mutants show decreased sterol levels (Fig. 6A) whereas *3betahsd/d1* shows increased sterol levels in root tissue (Fig. 9A). Together with the different subcellular localisations of RTN20, RTN19 and 3BETAHSD/D1 this raises the intriguing possibility that there might be additional layers of regulation by distributing an enzymatic function in different compartments and providing more localised sterol regulation.

RTN19 might be involved more in bulk sterol regulation throughout the ER network. RTN20 and 3BETAHSD/D1 activity can act more specifically in specific ER subdomains due to differing sterol requirements with specific biophysical properties or interaction with specific partners^49^. RTN20 and 3BETAHSD/D1 sterol regulation can also be linked to ERES formation and/or COPII vector fission in ER domains. Given a lack of growth phenotypes in the various single and the *rtn19/3betahsd/d1* double mutants^36^ each of the proteins might well compensate for the others. Therefore, to unravel the exact roles of these proteins, the putative enzyme activities and/or the search for their putative partners will have to be investigated. Furthermore we will have to take into account proteins involved in sterol biosynthesis and potential interactions (Supplementary Figure S8) and metabolic links between those and RTN20.

Taken together RTN20 as well as RTN19 can be considered a specialised subclass of reticulons most likely not directly involved in ER tubulation and structure as shown for other reticulons, but in lipid regulation. The different subcellular localisations of RTN20, RTN19 and 3BETAHSD/D1 might indicate additional layers of regulation and localised sterol regulatory hubs or factories. It will be of interest to see if other reticulons with additional N-terminal domain (RTN17, 18, 21) behave more like RTN20 and 19 or have again a completely different function in plant development.

## Methods

### Bioinformatics analysis

The functional domains of the reticulon family of proteins from *Arabidopsis thaliana* were analysed. The protein ATG numbers were searched for in TAIR and a protein BLAST in the *A. thaliana* database done to ensure all 21 sequences were correct and any splice variants of the proteins identified. A total of 35 sequences were obtained and phylogenetic analysis was performed with a one-click analysis using phylogeny.fr^50,51^. For phylogenetic analysis protein BLAST of RTN19 and 20 was performed within the yeast and human databases to identify homologous proteins. The sequences obtained were then used to search for additional arabidopsis proteins by doing a protein BLAST in the *A. thaliana* database. Phylogenetic analysis of the identified sequences was done using a phylogeny.fr one-click analysis, with the RTN17, 18 and 21 proteins used as outgroup.

Membrane topology with hydrophobic membrane-spanning regions was analysed using TOPCONS^52^.

### Cloning of expression plasmids

Primers were obtained from Eurofins Genomics. Q5 high-fidelity DNA polymerase (New England Biolabs) was used for all polymerase chain reaction reactions. Genes of interest were cloned into the modified binary vector pB7WGF2 containing an N-terminal or pB7FWG containing a C-terminal green fluorescent protein (GFP) and pB7RWG2 or pB7WGR2 for the red fluorescent protein (mRFP)^53^ using Gateway technology (Invitrogen).

### Plant material and transient expression in tobacco leaf epidermal cells

For *Agrobacterium*-mediated transient expression, 5-week-old tobacco (*Nicotiana tabacum* SR1 cv Petit Havana) plants grown in the greenhouse were used. Transient expression was carried out according to Sparkes *et al*. 2006^54^.

In brief, each expression vector was introduced into the *Agrobacterium* strain GV3101 by heat shock transformation. Transformant colonies were inoculated into 5 ml of YEB medium (5 g/l beef extract, 1 g/l yeast extract, 5 g/l sucrose and 0.5 g/l MgSO_4_·7H_2_O) with 50 μg/ml spectinomycin and rifampicin to keep the selection pressure up. The bacterial culture was incubated overnight in a shaker at 180 rpm 25 °C. 1 ml of the bacterial culture was pelleted by centrifugation at 2200 *g* for 5 min at room temperature. The resulting pellet was washed twice with 1 ml of infiltration buffer (50 mM MES, 2 mM Na_3_PO_4_·12H_2_O, 0.1 mM acetosyringone and 5 mg/ml glucose) and then resuspended in 1 ml of infiltration buffer. The bacterial suspension was diluted to a final OD_600_ of 0.1 and carefully pressed through the stomata on the lower epidermal surface using a 1 ml syringe.

Transformed plants then were incubated in a growth cabinet at 22 °C for 48 h. Samples from transient expression and transformed arabidopsis plants (cotyledons) were imaged using a 100×/1.46 NA oil immersion objective on a Zeiss LSM880 equipped with an Airyscan detector. For imaging of the GFP–RFP combinations, samples were excited using 488 and 543 nm laser lines in multi-track mode with line switching. Images were edited using the ZEN image browser.

### FRET-FLIM Data Acquisition

Tobacco epidermal leaf samples of infiltrated tobacco plants were excised, and FRET-FLIM data capture was performed according to Schoberer and Botchway^55^ using a two-photon microscope at the Central Laser Facility of the Rutherford Appleton Laboratory. GFP and mRFP expression levels in the plant samples within the region of interest were confirmed using a Nikon EC2 confocal microscope with excitation at 488 and 543 nm, respectively. A 633-nm interference filter was used to minimize the contaminating effect of chlorophyll autofluorescence emission. A two-photon microscope built around a Nikon TE2000-U inverted microscope was used with a modified Nikon EC2 confocal scanning system to allow for multiphoton FLIM^56^. 920 nm laser light was produced by a mode-locked titanium sapphire laser (Mira; Coherent Lasers), producing 200-fs pulses at 76 MHz, pumped by a solid-state continuous wave 532-nm laser (Verdi V18; Coherent Laser). The laser beam was focused to a diffraction limited spot through a water-immersion objective (Nikon x60 VC; 360, numerical aperture of 1.2) to illuminate the specimen. Fluorescence emission was collected without descanning, bypassing the scanning system, and passed through a BG39 (Comar) filter to block the near-infrared laser light. Line, frame, and pixel clock signals were generated and synchronized using a fast microchannel plate photomultiplier tube as external detector (Hamamatsu R3809U). Linking these via a time-correlated single-photon-counting PC module SPC830 (Becker and Hickl) generated the raw FLIM data. Data were analysed by obtaining excited-state lifetime values of a region of interest. Calculations were made using SPC Image analysis software version 5.1 (Becker and Hickl). The distribution of lifetime values within the region of interest was generated and displayed as a curve. Only values with a χ^2^ between 0.9 and 1.2 were taken into account. The median lifetime value in the region of interest was taken to generate the range of lifetimes per sample. At least three nuclei from a minimum of three independent biological samples per protein-protein combination were analysed, and the average of the ranges was taken.

### Reverse transcriptase-PCR

RNA was isolated using TRIzol® (Thermo Fisher Scientific) according to the manufacturer’s instructions. The AMV First Strand cDNA Synthesis Kit (New England Biolabs) was used according to the manufacturer’s instructions to create cDNA for wild type arabidopsis as well as for *rtn20* and *rtn6* mutants. The resulting cDNA was probed with primers for full-length products of RTN20 and RTN6, respectively using Q5® High-Fidelity DNA Polymerase (New England Biolabs).

### Lipid analysis

Lipids from roots and leaves of two week-old *Arabidopsis* seedlings (wild type and mutant lines) were extracted by grinding the tissues in a mixture of chloroform/methanol (2:1, v/v) at room temperature. Then lipid extracts were washed two times with 9‰ NaCl (1/4 of the organic solvent volume). The organic solvent was then evaporated under N2 gas stream and lipid extracts were dissolved in an appropriate volume of chloroform/methanol (1:1, v/v) and used for HPTLC (High Performance Thin Layer Chromatography) and GC-MS (Gas Chromatography-Mass Spectrometry) analyses.

#### HPTLC

Phospholipids were analyzed by loading total lipids onto HPTLC plates (60F254, Merck, Darmstadt, Germany), which were developed in methyl acetate/n-propanol/chloroform/methanol/0.25% aqueous KCl (25:25:25:10:9, v/v) according to Heape *et al*.^57^. To isolate and quantify sterols, total lipids were loaded onto HPTLC plates developed with hexane/ethylether/acetic acid (90:15:2, v/v) as in Laloi *et al*.^58^. Lipids were identified by co-migration with known standards and quantified by densitometry analysis (Macala *et al*., 1983) using a TLC scanner 3 (CAMAG, Muttenz, Switzerland) as described in Laloi *et al*.^58^.

#### GC-MS

For sterol MS analysis, the internal standard α‐cholestanol (0.02 mg) was added in 0.1 ml of lipid extract and the solvent was evaporated under N2 gas stream. A saponification step was performed by adding 1 ml of ethanol and 0.1 ml of 11 N KOH and incubating it for 1 h at 80 °C. After the addition of 1 ml of hexane and 2 mL of water, the sterol-containing upper phase was recovered, and the solvent was evaporated under N2 gas stream. Sterols were trimethylsilylated by N,O‐bis(trimethylsilyl) trifluoroacetamide (BSTFA)-trimethylchlorosilane for 15 min at 100 °C. After complete evaporation of BSTFA under N2 gas, derivatized sterols were dissolved in 0.1 ml of hexane and analyzed by GC-MS. GC-MS was performed using an Agilent 6850 gas chromatograph and coupled MS detector MSD 5975-EI (Agilent). An HP-5MS capillary column (5% phenyl-methyl-siloxane, 30-m, 250-mm, and 0.25-mm film thickness; Agilent) was used with helium carrier gas at 2 ml/min; injection was done in splitless mode; injector and mass spectrometry detector temperatures were set to 250 °C; the oven temperature was held at 50 °C for 1 min, then programmed with a 25 °C/min ramp to 150 °C (2-min hold) and a 10 °C/min ramp to 320 °C (6-min hold).

### EM fixation and data acquisition

Arabidopsis root segments were fixed in 1% glutaraldehyde and 1% paraformaldehyde in 0.1 M sodium cacodylate buffer (pH 6.9) for 60 min, washed in buffer and poststained for 12 hr in a mixture of zinc iodide and 1% osmium tetroxide^59^, followed by ethanol dehydration and embedding in Spurr resin (hard). Resin blocks were mounted onto 3View stubs with conductive epoxy glue (Chemtronics) and left to harden overnight. Blocks were trimmed and sectioned with a Gatan 3View system and SBFSEM images were collected with a Zeiss Merlin Compact field emission SEM. Slice thickness was 50–70 nm and the block face imaged under variable pressure (20–55 pa) at 3–4 keV with a pixel dwell time of 3 µs and pixel size of 4–6.2 µm. Initial image handling and alignment was carried out using *etomo* (IMOD, Boulder, Colorado; ref.^60^). Segmentation and reconstructions was achieved using Amira (Version 6.2, FEI, Eindhoven).

### Data availability

The authors declare that the data supporting the findings of this study are available within the paper and its supplementary information files.

### Accession numbers

Sequence data for the genes mentioned in this article can be found in the GenBank/EMBL databases using the following accession numbers: AT4G23630 (RTN1); AT4G11220 (RTN2); AT1G64090 (RTN3); At3g61560 (RTN6); At2g20590 (RTN17); At4g28430 (RTN18); At2g26260 (RTN19, 3BETAHSD/D2); At2g43420 (RTN20); At5g58000 (RTN21); AT1G47290 (3BETAHSD/D1).

## Electronic supplementary material

Supplementary Dataset 1
