# Supplementary material for: The odd one out: Arabidopsis reticulon 20 does not bend ER membranes but has a role in lipid regulation
Source: Sci Rep. 2018 Feb 2;8:2310. doi: 10.1038/s41598-018-20840-0 (PMC5797236; doi:10.1038/s41598-018-20840-0)
Supplement: Supplementary file 1 — Supplementary Dataset 1 [file 41598_2018_20840_MOESM1_ESM.pdf]

Title: The odd one out: *Arabidopsis* reticulon 20 does not bend ER membranes but has a role in lipid regulation

Verena Kriechbaumer, Lilly Maneta-Peyret, Laetitia Fouillen, Stanley W Botchway, Jessica Upson, Louise Hughes, Jake Richardson, Maike Kittelmann, Patrick Moreau, Chris Hawes

Supplementary figures:

Supplementary Figure S1:

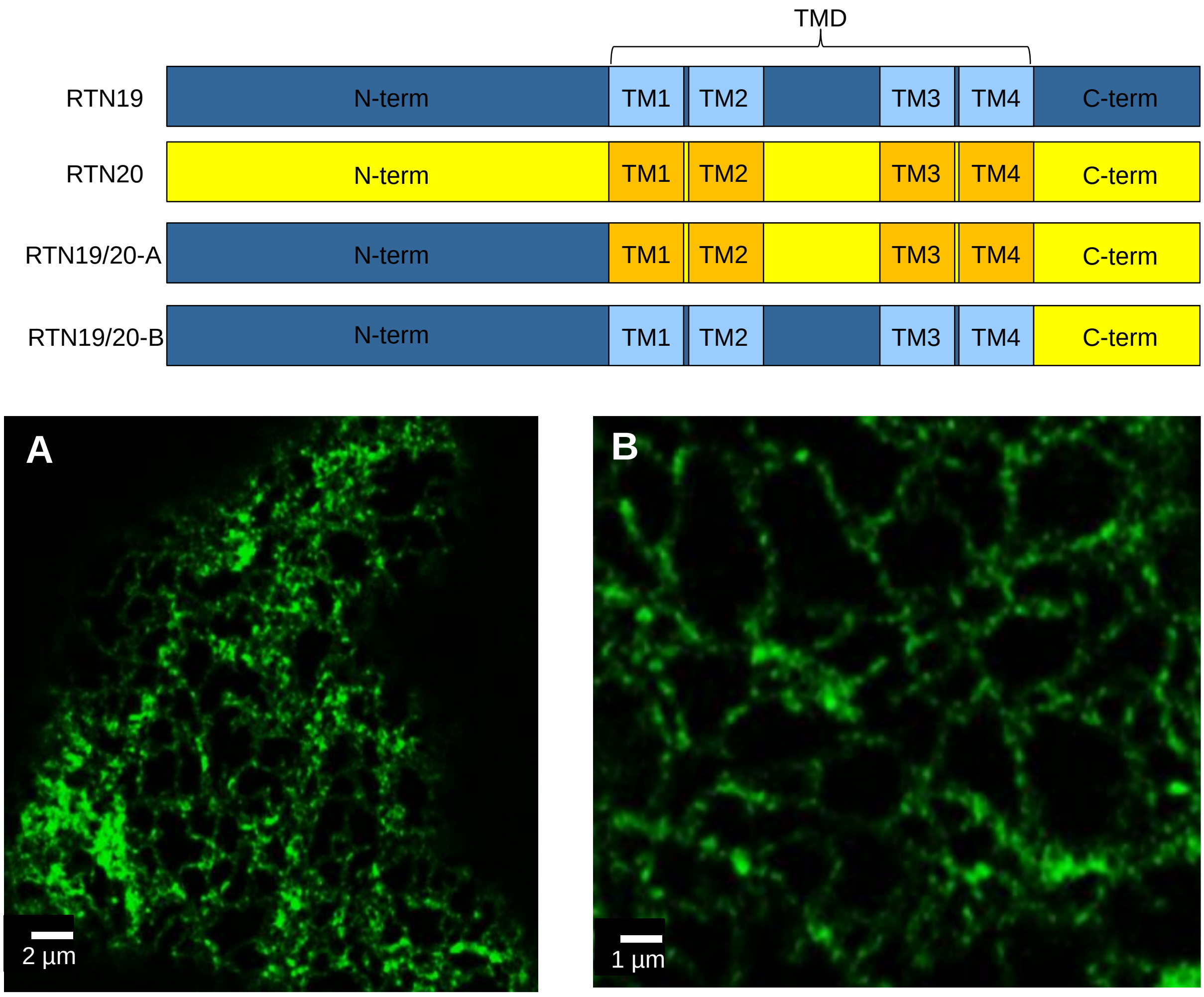

Supplementary Figure S1: The C-terminal domain of RTN20 changes the location of RTN19. Protein schemata: Two different chimeric protein fusions between RTN20 and RTN19 have been prepared (RTN19/20-A and RTN19/20-B). RTN19 sequence is shown in blue, RTN20 sequence in yellow. N- and C-terminal regions and transmembrane regions (TM) are indicated. (A) Confocal image of GFP fused to RTN19/20-A and (B) confocal image of GFP fused to RTN19/20-B. Both constructs display a punctuate localisation more similar to RTN20 than RTN19.

Supplementary Figure S2:

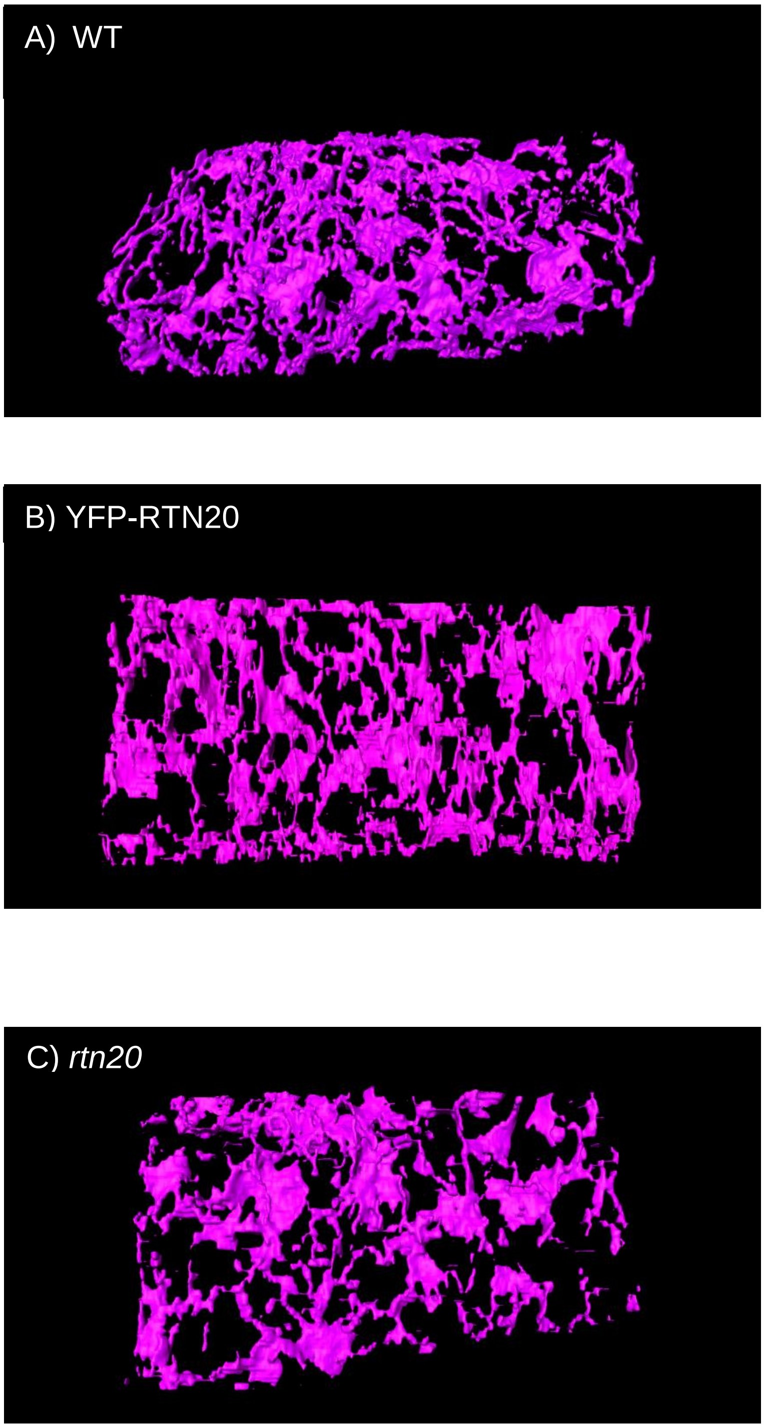

Supplementary Figure S2: 3D reconstructions of cortical ER in roots of (A) wildtype arabidopsis, (B) RTN20 overexpression lines and (C) *rtn20* mutants.

ER in arabidopsis root tip cells was selectively stained using the zinc iodide osmium tetroxide impregnation technique and reconstructed in 3-D by serial block face scanning electron microscopy. No major differences were observed in ER structure between wild type, RTN20 overexpression lines and *rtn20* mutants.

Supplementary Figure S3:

A)

RTN20 + (-)

RTN20 + RTN1

RTN20 + RTN2

RTN20 + RTN3

RTN20 + RTN19

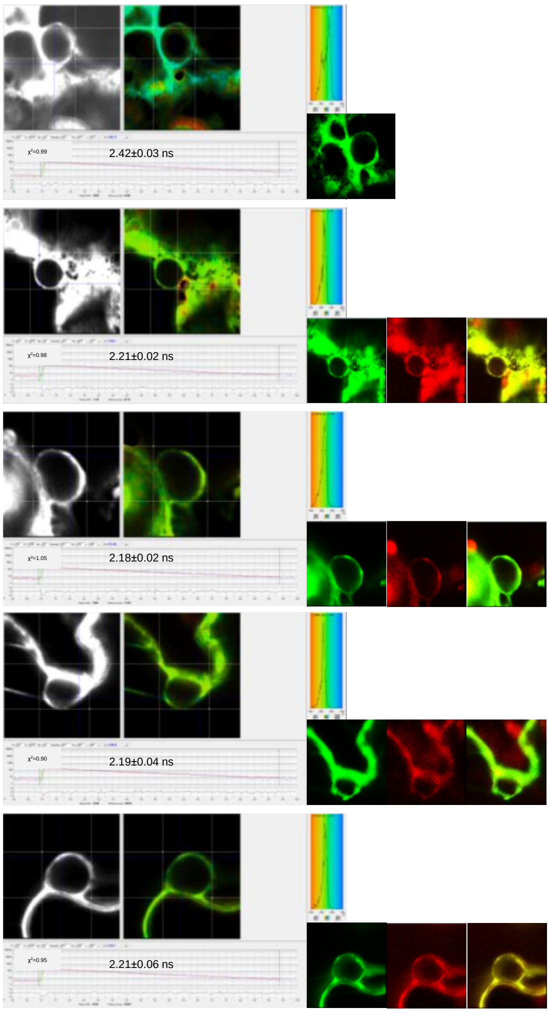

B)

Donor (GFP)	Acceptor (mRFP)	Average [ns]	SE	Δ [ns]
RTN20	(-)	2.4	0.03	0.0
	RTN1	2.2	0.02	0.2
	RTN2	2.2	0.02	0.2
	RTN3	2.2	0.04	0.2
	RTN19	2.2	0.06	0.2

Supplementary Figure S3: FRET-FLIM data for interactions

A) Representative FRET-FLIM data for interactions tested with GFP-RTN20 as donor proteins. Interactions are shown for RFP-RTN1, RFP-RTN2, RFP-RTN3, and RFP-RTN19. Corresponding confocal images with the GFP constructs in green and tested interacting proteins in red are shown on the right hand side.

B) The table shows the fluorescence lifetimes in FRET-FLIM analysis. Donor and acceptor protein constructs are indicated together with the average fluorescence lifetime (in ns) for the donor fluorophore and the Standard Error (SE) for each combination. The reduction in fluorescence lifetime of the donor is indicated (Δ). It was shown previously that a reduction in excited-state lifetime of 0.2 ns is indicative of energy transfer (Stubbs et al., 2005). For each combination, at least two biological samples with a minimum of three technical replicates were used for the statistical analysis.

Supplementary Figure S4:

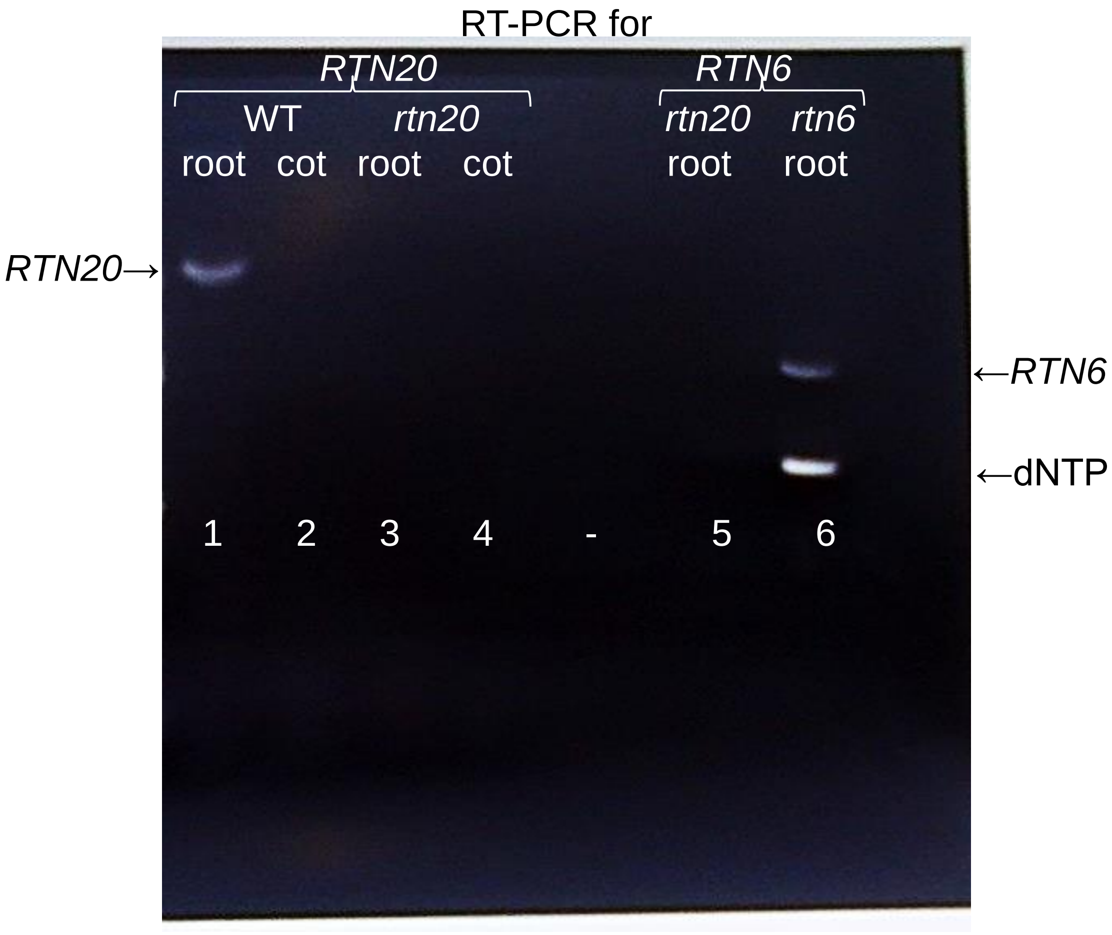

Supplementary Figure S5: Uncropped image for Figure 7.

Supplementary Figure S5:

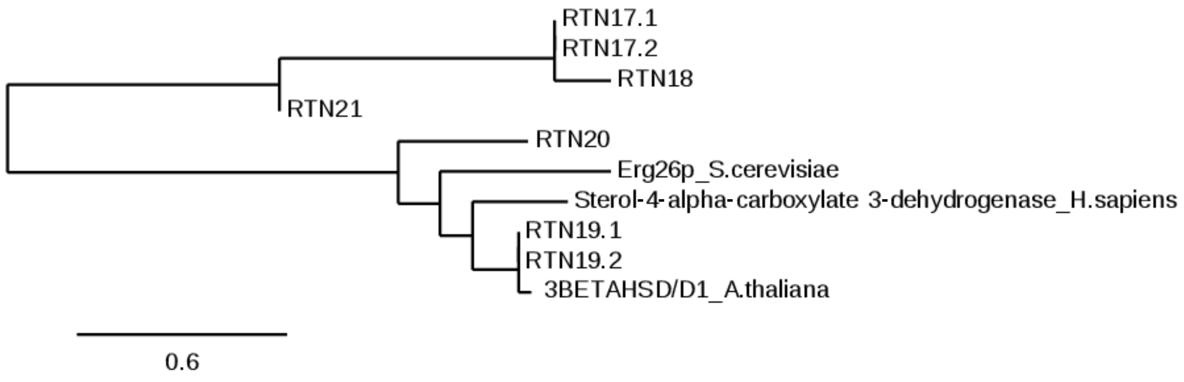

Supplementary Figure S5: Phylogenetic analysis of RTN20 homologue proteins.

A phylogenetic analysis with reticulon proteins featuring an additional N-terminal domain (RTN17-21) together with the closest mammalian homologues is shown. Bar for bootstrap value is shown

Supplementary Figure S6:

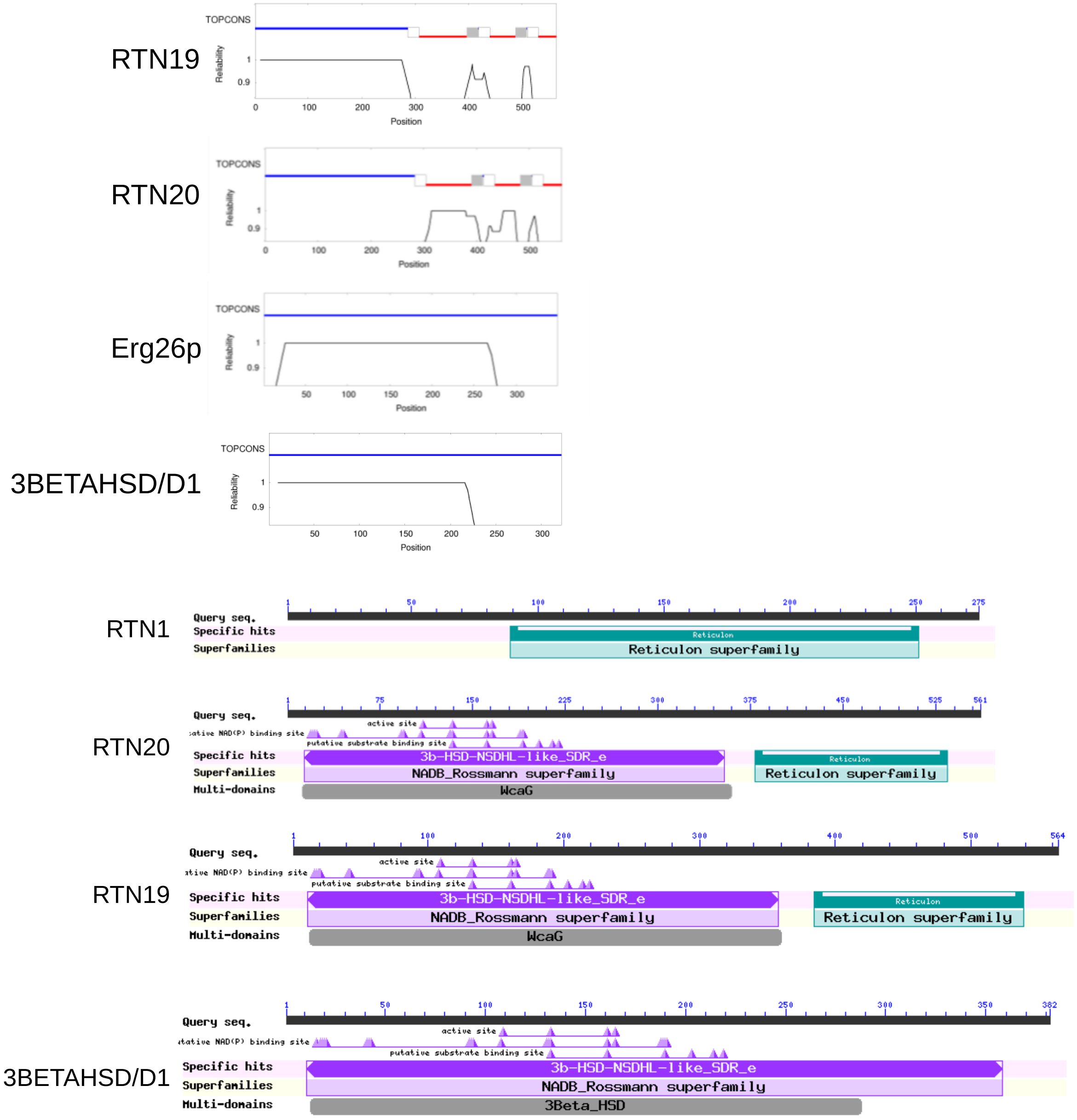

Supplementary Figure S6: Transmembrane domain (TMD) and protein domain predictions.
Top: RTN19 and RTN20 are predicted to feature the reticulon-typical four transmembrane domains (TMD) at the C-terminus. Unlike for the reticulon proteins 19 and 20, no transmembrane domains were predicted for the yeast homologous protein Erg26p and the arabidopsis protein 3BETAHSD/D1.
Bottom: Protein domain predictions for RTN1, 20, 19 and 3BETAHSD/D1. In addition to the reticulon domain in RTN1, RTN19 and RTN20 feature an N-terminal domain prediction (3b-HSD-NSDHL-like_SDR_e). 3BETAHSD/D1 is lacking the reticulon domain but features the N-terminal domain like RTN19 and RTN20.

Supplementary Figure S7:

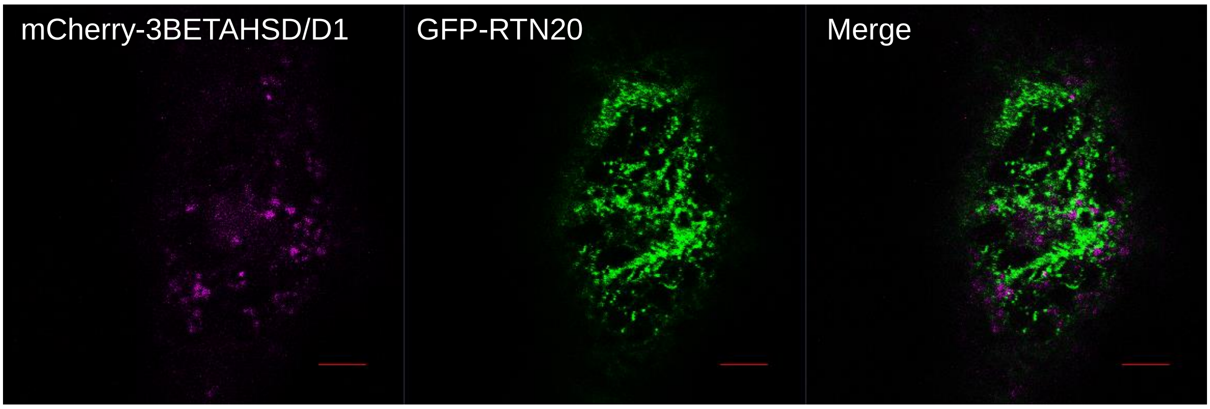

Supplementary Figure S7: Confocal image of 3BETSHSD/D1 and GFP-RTN20. 3BETAHSD/D1 tagged with the fluorescent protein mCherry does not colocalise with GFP-RTN20 punctuate structures.

Supplementary Figure S8:

A)

Protein	Gene ID	Function
DWF1	At3g19820	delta24 sterol reductase
DWF5	At1g50430	7-dehydrocholesterol reductase
DWF7	At3g02580	catalyzes delta7 sterol C-5 desaturation step, lathosterol oxidase
SMO2	At1g07420	sterol 4-alpha-methyl-delta7sterol-4alpha-methyl oxidase
SMT1	At5g13710	controls the level of cholesterol in plants, sterol 24-C-methyltransferase activity
SMT2	At1g20330	sterol-C24-methyltransferases involved in sterol biosynthesis, 24-methylenesterol C-methyltransferase
Cyp710A1	At2g34500	C-22 desaturase, catalyze in the presence of NADPH the conversion of β-sitosterol to stigmasterol

B)

	RTN20 (561 aa)	RTN19 (564 aa)	3BETAHSD/D1 (382 aa)
RTN20	561 in 561	220 in 525	162 in 349
RTN19	220 in 525	564 in 564	314 in 379
3BETAHSD/D1	162 in 349	314 in 379	382 in 382
DWF1	7 in 25	12 in 31	14 in 35 aa
DWF5	6 in 16	13 in 41	10 in 28
DWF7	10 in 38	4 in 6	No significant similarity
SMO2	18 in 72	8 in 24	10 in 32
SMT1	10 in 32	No significant similarity	3 in 7
SMT2	5 in 9	10 in 47	14 in 65
Cyp710A1	6 in 11	10 in 23	10 in 22

Supplementary Figure S8: Sterol biosynthetic proteins.

A) Arabidopsis proteins involved in sterol biosynthesis are listed with their gene ID and function (TAIR annotation).

B) The percentage of amino acid (aa) identity is given for the length of sequence that could be aligned between each pair (number of identical amino acids in total length of aligned sequence). The total length in amino acids is given for RTN20, RTN19 and 3NETAHSD/D1. Data show that only RTN20, RTN19 and 3BETAHSD/D1 show significant similarities. whereas DWF1, DWF5, DWF6 SMO2, SMT1, SMT2, and Cyp710A1 hardly align with RTN20, RTN19 or 3BETAHSD/D1.
